# Handwriting fluency, latency, and kinematic in Portuguese writing system: Pilot study with school children from 3rd to 5th grade

**DOI:** 10.3389/fpsyg.2022.1063021

**Published:** 2023-01-12

**Authors:** Giseli Donadon Germano, Simone Aparecida Capellini

**Affiliations:** Learning Disabilities Investigation Laboratory, Department of Speech and Hearing Sciences, São Paulo State University “Júlio de Mesquita Filho” (UNESP), Marilia, São Paulo, Brazil

**Keywords:** learning, handwriting, academic competencies, evaluation, social vulnerability

## Abstract

Studies have referred to the interaction between orthographic and motor aspects during the production of handwriting. However, studies with Brazilian Portuguese are still lacking. Hence, the aim of this study was to compare orthographic regularity, based on the Portuguese writing system, in high (HF) and low (LF) frequency words, in relation to latency and kinematic variables in students from the 3rd to the 5th grade of elementary school. This is a cross-sectional pilot study, with a convenience sample of 95 children participated in this study, from 3rd to 5th grade level attending a state elementary school. All were submitted to the following procedures of computerized evaluation of handwriting and submitted to the task of writing 15 HF and 15 LF words, selected according to the frequency criteria and classified according writing coding rule. Results indicated that for HF words, there was a decrease in writing and disfluencies production time, for all coding rules, from 3rd to 5th grade. However, for LF words, the more unpredictable orthographic affect production duration time, movement fluency, and students became more dependent on the use of gaze to check spelling aspects. This study revealed that lexical and sub-lexical activation affected motor production. For HF and LF words, lexical and sublexical process favored motor programming. However, for LF words, despite the maturation and school progression for the motor planes, there was an increase in latency time and in the need to search for word information, measured by the gaze variable for words with greater irregularity. This study has provided some evidence that linguistic variables such as orthographic regularity and word familiarity affect handwriting performance in Brazilian Portuguese written language.

## 1. Introduction

Learning to write is considered a linguistic skill that involves motor and orthographic aspects (Kandel and Perret, 2015a,b; Germano and Capellini, 2019). Recent models on the production of handwriting suggest that spelling processes modulate the timing of motor processes (Roux et al., 2013; Kandel and Perret, 2015a). Thus, for the authors, the central and peripheral processes interact during the production of handwriting, that is, there is a dynamic interaction between the processes, and the production of movement can be influenced by variables that regulate the orthographic process, such as frequency and lexicality (Roux et al., 2013). Ellis (1988) described that this is a cognitive neuropsychology approach, which central processes include semantic, syntactic and other sentence-level operations, along with those processes responsible for either retrieving from memory the spelling of a familiar word or assembling from sound a plausible spelling for an unfamiliar word or non-word. The end-product of these central processes is an abstract graphemic representation of words as letter strings. Peripheral writing processes translate that abstract graphemic representation into a range of possible output modes, including handwriting, typing, and spelling aloud.

In addition, central processes refer to spelling retrieval the activate information on the letter components of a word from orthographic long-term memory (Kandel et al., 2017). As mentioned by Purcell et al. (2011), spelling-specific central processes are usually identified as: orthographic long-term memory (the orthographic lexicon); phoneme–grapheme conversion; and orthographic working memory (the graphemic buffer). The authors also report that there is an interaction within the central processes, namely between the orthographic working and long-term memories and between the long-term orthographic memory and the phoneme-grapheme conversion.

The motor aspects of letter production are modulated by peripheral processes that regulate movement execution (Van Galen, 1991). However, Purcell et al. (2011) state that, in terms of peripheral processes, it is generally assumed that there are multiple stages involved in going from the abstract letter’s representations in orthographic working memory to the correct ordering and execution of the effector-specific muscle movements required for expressing these letters. These peripheral processes generate written language in the major modalities of oral spelling, written spelling, or typing, i.e. peripheral processes of written language are involved in this spelling “format,” including allographic or letter-shape conversion, motor plans for producing the letter forms, specifying size and ordering of the strokes (Ellis, 1988). These motor plans or graphic motor planning processes refer to an abstract representation of the movement that is then converted in motor commands that are specific for each end effector (for example, right or left hand, foot, etc.). As actions are encoded in the central nervous system in terms that are more abstract than commands to specific muscles, details of motor implementation, such as stroke size or speed, may be left unspecified until the effector is known. Once the effector is known, adjustment can then be made for effector-specific, indicating muscle activation patterns to accomplish letter size, and so on, given the specific writing context—are then generated and these, when executed, will result in the written trace (Wing, 2000). Furthermore, according with Marcelli et al. (2013), hypothesized that acquiring new motor skills requires two phases, in which two different processes occur, being during the early stage (spatial sequence is associated to the motor task in visual coordinates, i.e., the sequence of points to reach in order to generate the pencil trace) and during the late, i.e., automatic phase (sequence of motor commands in motor coordinates is acquired and comes to be executed as a single behavior).

Studies have demonstrated that writing can be produced by means of two separate routes, namely the lexical route and the sublexical route. In the lexical route, the orthographic form of the word is recovered as a whole from words stored in the long-term memory (Kandel and Valdois, 2006). Such lexical representations are influenced by the frequency of the words, in that the high-frequency words (HF) tend to be accessed with greater facility than the low-frequency words (LF; Bonin et al., 2016). The sublexical route applies the phonology-to-orthography conversion rules permitted by the language. This route is mostly used for the codification of non-familiar or non-words, although this route can also be used in parallel during the writing process (Coltheart and Rastle, 1994; Delattre et al., 2006; Afonso et al., 2018). It is important to highlight that both routes interact during the writing process, in that the information is manipulated and maintained in the orthographic working memory, in which the abstract graphemic units are maintained for subsequent production (Kandel et al., 2017; Afonso et al., 2020). In addition, Döhla et al. (2018) reported that during writing, working memory is important as it allows the maintaining and manipulating of phonological information in order to build orthographic representations of writing, from the establishment of the phoneme-grapheme conversion, linked with semantic information.

However, the interaction between spelling and motor processes can be restricted. Most grapho-motor gestures require extreme control and close sensory guidance (Mojet, 1991). Motor control is cognitively very demanding, as child concentrates on producing the correct shapes and connecting the letters between them. With practice there is a progressive learning of sensory-motor maps or motor programs (Teulings et al., 1983) that are stored in long-term memory. The use of this maps facilitates rapid access, diminishing use of sensory feedback and increases movement speed. This entails a long process that ends around 10–11 years old. At this period movement production is fast, implicit and automatic (Halsband and Lange, 2006). With neuro-motor maturation, grapho-motor skills become automatic (Van Galen et al., 1993) and children can use their cognitive resources for the other components of writing, such as spelling, sentence construction and text elaboration (Maggio et al., 2012; Pontart et al., 2013). The literature reports that the relation between the orthographic and motor aspects during the production of handwriting has been described in various languages with differing opacities and transparency; notably languages with greater opacity, such as French (Kandel and Perret, 2015a,b) and those with greater transparency such as Spanish (Afonso et al., 2015, 2018; Afonso and Álvarez, 2019). However, studies with Brazilian Portuguese are still lacking. The writing system for Brazilian Portuguese is characterized by orthography-to-phonology transparency for reading but is opaque in terms of writing (phonology-to-orthography opacity; Scliar-Cabral, 2003a,b). To be able to write the schoolchild needs to understand that the writing system comprises three types of relationships between the phonemes and graphemes of the alphabet. The first type of relationship is the biunivocal correspondence, consisting of the relationship between sounds and letters, or that is, the conversion of phonemes into graphemes independently from their context (regular words). The second type is the sound-letter relationship in which a letter represents various phonemes and also in which a phoneme can be represented by different graphemes according to their location within the word, or that is, there can be a certain predictability for conversion of phonemes into graphemes, depending on their position and/or their phonetic context (for example, the phoneme/k/can be written with the graphemes [c] or [qu]; irregular words). The third possibility for the type of relationship between phonemes and letters presents a situation of concurrence with a totally arbitrary relationship between the orthographic system and the phonological system, or that is, competing alternatives (for example, the phoneme/s/can be represented by the letters S/C/SS/SC/Ç/SÇ/XC). Regarding performance of children in coding writing, a greater difficulty is noted, since there are significant irregularities that need to be taught then systematized and memorized by children (irregular, and more unpredictable type of words; Scliar-Cabral, 2003a,b).

Roux et al. (2013) found that the syllabic position with the irregularity may affect triggering the production of writing, i.e., about the interaction between central and peripheral processing. The authors observed that if the orthographic irregularity is in the first syllable, the cascade effect is immediately performed. However, if it is second or final position of the word, the effect becomes permanent until the irregularity is achieved. In another words, Delattre et al. (2006) examined whether writing latencies and durations were affected by central processes at the lexical (word frequency) and sublexical levels (orthographic regularity). The cascaded view predicted that durations – which reflect peripheral processing – was affected by these variables because orthographic retrieval still operate after the initiation of the writing movements. The outputs are integrated either at the graphemic buffer, or at the grapheme level as claimed by recent implementations. With irregular words, if it is still not entirely solved when writing begins, it continues to be processed on-line, that is, until the irregular spelling conflict is resolved, regardless of syllable position in the word. This slows down the processing of the whole movement, increasing durations of irregular words with respect to regular ones. Thus, the authors concluded that both frequency and spelling regularity produce different effects of cascade, as verified by Roux et al. (2013). In this way, this study chose to verify kinematic variables and latency of HF and LF words, maintaining the activation of the same motor program (i.e., B). There was no comparation between HF and LF words because we would be talking about different activation processes, which was not the focus of this study.

In this way, it’s expected that with automaticity, orthographic knowledge can be processed in parallel to movement production. Aspects such as frequency and orthographic regularity are related to the central process of writing production, since they can generate different kinematic processes [peripheral processes (Kandel and Perret, 2015a,b)].

Thus, this study is justified by the idea that writing fluency is an important aspect of writing development in the first grades so that other advanced skills can be achieved, such as text production. Kim et al. (2018) defined writing fluency as efficient and automatic writing connected texts, with accuracy, speed, and straightforwardness that is writing fluency is efficiency and automaticity in writing text. According to information processing theory (LaBerge and Samuels, 1974), fluency is a developmental phenomenon, spanning several stages, including sublexical, lexical, and text or speech levels, and lower-level fluency is necessary to achieve fluency at a higher level. Also, Berninger et al. (2008) emphasis the key role of handwriting automation in their Simple View of Writing model, highlighting the importance of efficient and fluent execution of lower-level processes in order to execute higher level metacognitive processes in composing a text. There are still no studies investigating this issue in Brazil, justifying this pilot study.

In this way, we seek to measure the latency time, related to the time the children needed to prepare the movements to start writing a word. We also tried to evaluate kinematic variables, such as movement duration and fluency. Thus, the aim of this study was to compare orthographic regularity, based on the Portuguese writing system, in HF and LF frequency words, in relation to latency and kinematic variables in students from the 3rd to the 5th grade of elementary school.

## 2. Materials and methods

This study was conducted following approval by the Research Ethics Committee of the Faculty of Philosophy and Sciences of São Paulo State University “Júlio de Mesquita Filho” (UNESP), Marilia, São Paulo, Brazil, under number CAAE: 87368618.4.0000.5406. All participants signed the Free and Informed Consent Form.

### 2.1. Participants

This is a cross-sectional pilot study carried out before the pandemic. A convenience sample of 95 children participated in this study, from 3rd to 5th grade level attending a state elementary school. There were 27 children attending 3rd grade (mean age: 8 years and 7 months; standard deviation: 2.54), 37 children attending 4th grade (mean age: 9 years and 3 months; standard deviation: 2.29) and 31 children attending 5th grade (mean age: 10 years and 9 months; standard deviation: 2.61). They were all right-handed, according to the motor assessment by Rosa Neto (2002) and native Brazilian speakers. The selection of participants for this study was realized by nonprobability convenience sampling, or that is, they were selected according to those who were available for the proposed evaluations twice per week. Participant recruitment and data collection took place over 2 months, in the second educational semester, from July and December 2018. After approval and consent by the school board, students were invited to participate in the study. Participation was confirmed after presentation of the Free and Informed Consent Form signed by the child’s parent/guardian. The study protocol was approved by the Research Ethics Committee and all procedures followed the Helsinki Declaration. The inclusion criteria for the study were (a) ages 8–10 years-old and (b) teachers’ observations of good academic performance. The exclusion criteria for the study were students (a) with sensory (auditory and/or visual), cognitive or physical deficits; (b) who did not complete at least 80% of the assessment; and (c) voluntary withdrawal.

### 2.2. Procedure

The children were evaluated individually, in 3 to 4 sessions, with a maximum duration of 30 min. Most students performed the writing of the HF word list in one session, and the LF list in another session, following that order. If there was any intercurrence, such as the student being called back to the classroom by the teacher, a new session (third or fourth) was used. The procedures were performed before the covid-19 pandemic, between July and December 2018. All were submitted to the following procedures of *Computerized evaluation of handwriting* (Ductus software®; Guinet and Kandel, 2010). To perform the procedures described below, a notebook computer was used (adapted version; Germano, 2018; Germano and Capellini, 2019) coupled to a digitizing table (Intuos Pro Wacom Pen and Touch Tablet). The stimuli were presented in the center of the notebook screen (written in capital letters – Times New Roman size 18). An auditory signal and a fixation point (duration of 100 ms) preceded the presentation of the stimuli. The stimulus remained on the screen until the student had finished writing the word. The student was instructed to write the word on the graphics tablet as soon as it appeared on the notebook screen. All student performance writing capital letters. This choice was made based on Brazilian Educational System, which is based on the current literacy curriculum approach, which is related to whole language. As mentioned by Germano and Capellini (2019) one aspect to be considered in the Brazilian context is the absence of systematic teaching of the movements of writing letters and the changes that occurred in the mid-1980s, when the teaching of the letter-writing movements was relegated to a secondary plane and the aspects of language were emphasized instead. They were submitted to the task of writing 15 HF (mean number of occurrences = 69; median = 58, range = 28–131) and 15 LF (mean number of occurrences = 1.0; median = 1, range = 1.0) frequency words, selected according to the frequency criteria (Germano, 2018; Table 1).

**Table 1:** Absolute number of occurrences of HF and LF words.

	HF	*N*	LF	*N*
C1	Pato/duck	109	Bolha/Bubble	1
	Olho/eye	58	Dama/lady	1
	Nome/Name	52	Mapa/map	1
	Velha/old	31	Moto/motorcycle	1
	Vida/life	33	Tipo/type	1
C2	Lobo/Wolf	131	Flora/flora	1
	Gato/cat	111	Regra/rule	1
	Tempo/time	60	Ruga/wrinkle	1
	Mundo/world	55	Saga/saga	1
	Cama/bed	51	Flanco/flank	1
C3	Casa/house	121	Chance/chance	1
	Coisa/thing	85	Classe/class	1
	Gente/people	76	Concha/shell	1
	Bruxa/witch	34	Xadrez/chess	1
	Bicho/animal	28	Chifre/horn	1
	Mean	69	Mean	1
	Median	58	Median	1
	N: absolute occurrence number			

The words were taken from school vocabulary, composed of words extracted from Portuguese Language books from the 1st to the 5th grade level of Elementary Education of State of São Paulo (Germano and Capellini, 2011; Germano, 2018). Only disyllable nouns, of different syllabic complexities, regular and irregular words were included. The following classes of words were excluded: words in other languages, adverbs, adverbial phrases, prepositive phrases, adjectives, months of the year, numerals, augmentative or diminutive words, slang and words composed by juxtaposition words that present some diacritical signs and words with “ç.” The list formed had words of different syllabic complexities, regular and irregular words, randomized by frequency. For both HF and LF words were classified according writing coding rule of, Scliar-Cabral (2003a,b), being 5 words classified as rule C1 (Conversion of phonemes to graphemes regardless of context – Phonographic conversion is not determined by position or phonetic context, that is, there is no restriction on the grapheme assignment in 12 phonemes – that is, each phoneme can be represented only by a single grapheme, being a univocal phoneme-grapheme correspondence, 5 words were classified as rule C2 (Conversion of phonemes to graphemes depending on position and/or phonetic context – Phonographic conversion, in these rules, depends on how the phonemes are pronounced, for the choice of letters or graphemes that will represent them) and 5 words as rule C3 (Competitive alternatives – there is competitiveness for the same phonetic context, it is necessary to have a metalinguistic knowledge, especially semantics and morphology, which can help in choosing the letter or grapheme that will represent it; words are dependent on orthographic lexical memory). We analyzed four measures, described below.

- Latency referred to the time between word presentation and the moment the child started to write (pen pressure > 0).
- A measure of “gaze” was used, that is, the moment when the child stop their handwriting to search/looks up at the screen to confirm the information about the words. The elevations of the gaze were considered as a “landmark of the event,” being an option for the Researcher who can place a “mark” at any time on what the student produces. This marking was performed by the researcher by pressing the space key on the notebook keyboard, and later confirmed from the images recorded with a video camera, positioned so that it could capture eye movement.

Regarding information about kinematics aspects of motor production, we have measured movement duration and fluency:

- Writing word movement duration – referring the time the children took to write a complete word (summatory of each letter in a word, Movement duration – ms). The movement duration of a word was computed by summing up the time spent to draw each letter of the word, which was normalized with respect to the number of strokes that made up the letter, based on the criteria described in studies (Thibon, 2018; Thibon et al., 2018, 2019).
- Movement fluency is measured as the mean number of peaks of the absolute velocity profile per letter of a word. In particular, the total number of peaks is obtained by summing up the number of peaks counted in the absolute velocity profile of each letter of the word. For movement fluency, the sum was performed followed by the division of the number of letters, resulting in the average velocity of the word. It is noteworthy that the higher these values, the lower the movement fluency (disfluency; Lambert et al., 2011; Kandel and Perret, 2015a,b).

A stroke can be defined as a fundamental unit of handwriting movement, that is, a sequence of movement performed between two absolute velocity minima (Guinet and Kandel, 2010). For each letter, the calculation was considered from the contact of the pen on the digitizing table (pressure > 0), continuing until the end of the tracing (pressure = 0). We perform the calculation for each letter of each word, calculating the number of strokes presented in the segmentation of letters (Guinet and Kandel, 2010). So, we divided the values by the number of strokes in each letter, based on the criteria described in studies (Thibon, 2018; Thibon et al., 2018, 2019) for standardizing the difference in the types of strokes provided for in the different letters (for example the letter L has two strokes, while the letter B has five strokes).

### 2.3. Data analysis

We gathered, calculated, and presented descriptive statistics, including group means and standard deviations. Data analysis was performed with statistical analysis of the scores, using the SPSS (Statistical Package for Social Sciences) program. The ANOVA statistical test was used, verifying normal distribution with zero mean and constant variance per grade. A graphical analysis was also performed, and it was found that the data distribution of each measurement per grade is close to a normal distribution and that they have homoscedasticity. The value of *p* < 0.05 was considered significant and indicated by an asterisk (^*^).

## 3. Results

Table 2 shows the distribution of variables latency, movement duration, movement fluency and gaze in the comparison between groups for HF words.

**Table 2:** Comparison between the variables for groups for HF words.

Rule	Word	Group	Latency			Movement duration			Movement fluency			Gaze		
			Mean	SD	Value of *p*	Mean	SD	Value of *p*	Mean	SD	Value of *p*	Mean	SD	Value of *p*
C1	Pato/duck	3rd	3,443	1916	0.005^*^	1,331	641	0.019^*^	8.13	4.44	0.085	0.148	0.362	0.066
		4th	2,249	848		1,283	449		7.48	2.53		0.054	0.229	
		5th	2,310	1760		983	453		6.2	3.15		0	0	
	Olho/eye	3rd	2,413	1,181	0.145	1,360	884	0.014^*^	8.76	6.14	0.002^*^	0.111	0.32	0.171
		4th	1988	703		1,216	390		7.5	2.36		0.054	0.229	
		5th	1977	989		931	324		5.31	1.83		0	0	
	Nome/Name	3rd	2,360	1,372	0.13	1,160	593	0.002^*^	6.92	3.95	0.006^*^	0.111	0.32	0.171
		4th	1934	854		1,018	451		6.22	2.3		0.054	0.229	
		5th	1886	609		749	208		4.76	0.85		0	0	
	Velha/old	3rd	2,590	936	0.012^*^	1,336	502	0.003^*^	7.13	2.54	0.005^*^	0.111	0.32	0.04^*^
		4th	2006	1,234		1.262	414		6.8	1.83		0.189	0.397	
		5th	1821	658		990	254		5.6	1.02		0	0	
	Vida/life	3rd	2,529	1,404	0.028^*^	1,405	814	0.009^*^	7.4	4.21	0.017^*^	0.037	0.192	0.287
		4th	1978	897		1,149	482		6.29	3.05		0	0	
		5th	1777	943		946	278		5.07	1.36		0	0	
C2	Lobo/Wolf	3rd	3,398	2,199	0.417	9,695	4,843	0.46	9.36	1.44	0.001^*^	0.037	0.192	0.256
		4th	3,496	2,482		8,977	2,960		2.9	1.22		0.081	0.277	
		5th	2,854	1,320		8,451	3,597		2.37	1.62		0	0	
	Gato/cat	3rd	2099	762	0.191	1,417	591	<0.001^*^	8.64	3.4	<0.001^*^	0.037	0.192	0.287
		4th	1806	1,001		1,105	402		6.82	2.56		0	0	
		5th	1715	624		844	195		5.34	0.91		0	0	
	Tempo/time	3rd	2,418	1,258	0.021^*^	1,313	618	0.004^*^	8.19	3.72	0.006^*^	0.222	0.424	0.022^*^
		4th	2008	1,196		1,161	406		6.88	1.53		0.108	0.315	
		5th	1,616	653		927	225		6.24	1.11		0	0	
	Mundo/world	3rd	2,687	1,289	0.112	1,195	714	0.005^*^	7.53	5.16	0.029^*^	0.074	0.267	0.107
		4th	2004	1,108		1,130	535		6.96	2.96		0.135	0.347	
		5th	2,107	1,599		784	189		5.27	0.84		0	0	
	Cama/bed	3rd	2,956	1,574	0.007^*^	925	427	0.001^*^	5.94	2.58	0.007^*^	0.074	0.267	0.275
		4th	2,387	1896		1,026	360		6.17	1.65		0.027	0.164	
		5th	1,643	926		710	176		4.81	0.89		0	0	
C3	Casa/house	3rd	2,666	1798	0.995	1,067	567	0.007^*^	6.72	3.94	0.019^*^	0.037	0.192	0.595
		4th	2,676	1975		1.054	382		6.63	2.47		0.027	0.164	
		5th	2,712	1,600		770	246		4.96	1.35		0	0	
	Coisa/thing	3rd	2024	1,304	0.998	1,358	717	0.082	8.3	4.44	0.248	0.185	0.396	0.095
		4th	2014	1,025		1,223	455		7.86	2.91		0.108	0.393	
		5th	2032	1,279		1,021	557		6.77	3.57		0	0	
	Gente/people	3rd	1847	1,314	0.412	1,202	771	0.175	7.42	144.33	0.002^*^	0.222	0.424	0.064
		4th	2,216	1,207		1,145	469		7.18	1.9		0.054	0.229	
		5th	1921	1,051		939	475		6.39	2.56		0.065	0.25	
	Bruxa/witch	3rd	2,374	857	0.012^*^	1,263	602	0.001^*^	7.84	3.47	0.001^*^	0.111	0.32	0.171
		4th	1946	948		1,118	388		6.71	1.68		0.054	0.229	
		5th	1,650	892		853	195		5.56	0.81		0	0	
	Bicho/animal	3rd	3,157	2,608	0.003^*^	1,369	747	0.012^*^	8.15	4.67	0.013^*^	0.148	0.362	0.101
		4th	2071	909		1,222	545		7.34	3.32		0.027	0.164	
		5th	1786	737		948	221		5.63	1.11		0.032	0.18	

Anova test (^*^p < 0.05).

Regarding the HF words, Table 2 indicated that there was a difference between the groups for the Latency variable in relation to the rule words C1 (“pato/duck,” “velha/old,” “vida/life”), C2 (“tempo/time” and “cama/bed”) and C3 (“bruxa/witch” and “bicho/animal”). Regarding the movement duration, there was a difference between all the words from C1, to C2 (most, except the word “lobo/wolf”); and for C3 for the word “casa/house,” “bruxa/witch” and “bicho/animal.” Regarding the movement fluency, there was a difference for C1 for most words, except “pato/duck”; for C2 for all words; and for C3 for the word “casa/house,” “gente/people,” “bruxa/witch” and “bicho/animal.” In relation to gaze, there was a difference for C1 (“velha/old”) and C2 (“tempo/time”), with no difference for the words of C3. To better verify such differences, a comparison was made between the *p* values, in order to verify which groups presented the comparisons, based on Tukey’s Multiple Comparison (*post hoc*; Table 3).

**Table 3:** Comparison of *p* values for variables between groups for HF words.

Rule	Word	Group	Latency		Movement duration		Movement fluency		Gaze	
			3rd	4th	3rd	4th	3rd	4th	3rd	4th
C1	Pato/duck	4th	0.007^*^		0.925		0.728		0.273	
		5th	0.016^*^	0.985	0.03^*^	0.047^*^	0.08	0.265	0.055	0.626
	Olho/eye	4th	0.188		0.571		0.38		0.571	
		5th	0.196	0.999	0.013^*^	0.097	0.002^*^	0.047^*^	0.146	0.58
	Nome/Name	4th	0.196		0.413		0.542		0.571	
		5th	0.157	0.978	0.002^*^	0.036^*^	0.006^*^	0.057	0.146	0.58
	Velha/old	4th	0.057		0.742		0.757		0.563	
		5th	0.011^*^	0.725	0.004^*^	0.017^*^	0.006^*^	0.026^*^	0.344	0.03^*^
	Vida/life	4th	0.113		0.165		0.323		0.33	
		5th	0.026^*^	0.727	0.006^*^	0.287	0.013^*^	0.232	0.358	1
C2	Lobo/Wolf	4th	0.981		0.734		0.004^*^		0.663	
		5th	0.585	0.419	0.427	0.835	0.002^*^	0.958	0.764	0.228
	Gato/cat	4th	0.344		0.011^*^		0.012^*^		0.33	
		5th	0.187	0.894	<0.001^*^	0.032^*^	<0.001^*^	0.042^*^	0.358	1
	Tempo/time	4th	0.29		0.355		0.066		0.292	
		5th	0.015^*^	0.293	0.003^*^	0.075	0.005^*^	0.488	0.016	0.303
	Mundo/world	4th	0.112		0.876		0.78		0.622	
		5th	0.23	0.946	0.009^*^	0.02^*^	0.032^*^	0.102	0.525	0.087
	Cama/bed	4th	0.318		0.459		0.867		0.541	
		5th	0.005^*^	0.123	0.044^*^	0.001^*^	0.049^*^	0.007^*^	0.248	0.802
C3	Casa/house	4th	1		0.992		0.989		0.96	
		5th	0.995	0.996	0.019^*^	0.015^*^	0.041^*^	0.036^*^	0.598	0.725
	Coisa/thing	4th	0.999		0.624		0.882		0.616	
		5th	1	0.998	0.07	0.319	0.248	0.433	0.081	0.36
	Gente/people	4th	0.441		0.918		0.004^*^		0.077	
		5th	0.97	0.566	0.192	0.304	0.006^*^	0.999	0.123	0.989
	Bruxa/witch	4th	0.153		0.356		0.103		0.571	
		5th	0.009^*^	0.375	0.001^*^	0.028^*^	<0.001^*^	0.082	0.146	0.58
	Bicho/animal	4th	0.019^*^		0.531		0.599		0.122	
		5th	0.003^*^	0.733	0.01^*^	0.097	0.013^*^	0.089	0.167	0.996

Anova test, Tukey’s multiple comparison (*post hoc*; ^*^p < 0.05).

At Table 3, it was possible to observe that the difference was present in the comparison between 3rd and 5th grade students for the Latency variable, in relation to rules C1, C2 and C3. Regarding the movement duration, it was observed that there is a decrease in the duration time for the production of words, noticed between 3rd and 5th and 4th and 5th, for the words of C1, C2 and C3. For movement fluency, there is a decrease in disfluencies with the progression of schooling, especially for C1 words. Regarding the words C2 and C3, it was noted that there is a greater difference between 3rd and 5th grade students, suggesting that improved access to the motor and lexical plane for HF words occurs at the end of elementary school (5th grade level). Also, it is noted that the difference for the gaze variable occurred between students from 4th and 5th grade, as 4th grade students still needed to search for word’s characteristic on the notebook’s screen, suggesting that the orthographic lexicon was not formed for the words “velha/old” (C1) and “tempo/time” (C2). Despite being frequent words, the word “velha” (old) requires knowledge that a phoneme (/λ/) must be written by two letters (lh), while the word “tempo” (time) requires knowledge of the rule code |C2.16.2|, which indicates that the use of m (before/p/and/b/) or the letter n (before other consonants) as nasalization marks in the conversion of nasalized vowels at the end of syllables internal. Brazilian study has indicated such difficulties present in schoolchildren due to the lack of systematic teaching of the phoneme-grapheme conversion mechanism and the explicit explanation of spelling rules at school (Chiaramonte and Capellini, 2022). Table 4 shows the distribution of latency variables, writing duration movement, writing fluency movement and gaze in the comparison between groups for LF.

**Table 4:** Comparison between the variables for groups for LF words.

Rule	Word	group	Latency (ms)			Movement duration			Movement fluency			Gaze		
			Mean	SD	Value of *p*	Mean	SD	Value of *p*	Mean	SD	Value of *p*	Mean	SD	Value of *p*
C1	Bolha/Bubble	3rd	3,707	2025	<0.001^*^	1,206	607	0.493	7.38	2.76	0.318	0.148	0.362	0.209
		4th	2,285	1,355		1,149	342		6.98	1.27		0.054	0.229	
		5th	1987	918		1,066	405		6.55	2.19		0.032	0.18	
	Dama/lady	3rd	2004	1,113	0.441	869	437	0.073	5.27	1.73	0.299	0.074	0.267	0.275
		4th	2,266	1754		879	367		5.28	1.51		0.027	0.164	
		5th	1829	1,168		702	173		4.79	0.86		0	0	
	Mapa/map	3rd	2,364	1,406	0.062	883	376	0.093	5.44	1.8	0.282	0.111	0.32	0.088
		4th	1919	909		889	338		5.51	1.59		0	0	
		5th	1728	746		728	264		4.96	1.04		0.032	0.18	
	Moto/motorcycle	3rd	2,168	951	0.271	1,016	538	0.035^*^	6.45	2.87	0.055	0.074	0.267	0.077
		4th	2011	958		1,015	593		6.62	3.27		0	0	
		5th	1785	784		738	184		5.16	1		0	0	
	Tipo/type	3rd	2,749	1,364	<0.001^*^	1,011	347	0.187	2.54	2.38	0.218	0	0	0.124
		4th	1700	736		971	215		2.54	1.21		0	0	
		5th	1700	820		892	188		2.54	1.19		0.065	0.25	
C2	Flora/flora	3rd	3,465	2,251	<0.001^*^	1,104	437	0.006^*^	7.08	2.08	0.006^*^	0.222	0.506	0.096
		4th	1961	768		1,178	493		7.3	2.25		0.243	0.495	
		5th	2077	1,279		865	193		5.87	0.94		0.032	0.18	
	Regra/rule	3rd	2,533	1703	0.233	1,080	494	0.002^*^	7.03	2.48	0.013^*^	0.074	0.267	0.186
		4th	2,155	951		1,080	381		6.94	2.01		0.108	0.315	
		5th	1989	1,003		792	161		5.72	0.99		0	0	
	Ruga/wrinkle	3rd	3,060	2,269	0.001^*^	974	450	0.034^*^	6.21	2.53	0.02^*^	0.074	0.267	0.104
		4th	2,110	1,099		932	379		5.89	2.2		0.162	0.442	
		5th	1,560	630		745	216		4.8	1.01		0	0	
	Saga/saga	3rd	2,494	1,180	0.019^*^	820	249	0.01^*^	5.3	1.38	0.073	0.037	0.192	0.595
		4th	1922	681		877	325		5.48	1.49		0.027	0.164	
		5th	1891	833		685	143		4.77	0.81		0	0	
	Flanco/flank	3rd	3,649	2,312	0.032^*^	1,398	558	0.021^*^	8.4	2.92	0.038^*^	0.556	0.698	0.908
		4th	2,793	2058		1,389	492		8.17	2.06		0.486	0.768	
		5th	2,324	1,150		1,098	360		7.05	1.35		0.484	0.626	
C3	Chance/chance	3rd	2,680	1,488	0.217	1,429	761	0.005^*^	9.22	4.76	0.005^*^	0.667	0.62	0.053
		4th	2,304	1,147		1,228	471		7.77	2.47		0.324	0.475	
		5th	2,144	883		976	227		6.5	1.27		0.484	0.57	
	Classe/class	3rd	2,752	1,512	0.139	1,361	804	0.003^*^	8.81	4.47	0.002^*^	0.63	0.492	0.004^*^
		4th	2061	1,013		1,110	255		7.32	1.24		0.324	0.475	
		5th	2,298	1,586		927	234		6.32	1.25		0.226	0.425	
	Concha/shell	3rd	2,420	1,682	0.216	1,379	804	0.012^*^	8.62	4.7	0.045^*^	0.481	0.509	0.002^*^
		4th	2,714	1784		1,129	301		7.64	1.9		0.162	0.442	
		5th	2040	1,125		991	239		6.69	1.53		0.097	0.301	
	Xadrez/chess	3rd	2,928	2,563	0.043^*^	1,356	582	0.013^*^	8.2	3.6	0.065	0.778	0.641	0.056
		4th	2,165	935		1,176	466		7.79	2.57		0.541	0.558	
		5th	1917	842		1,008	189		6.69	0.9		0.419	0.502	
	Chifre/horn	3rd	2,341	1,496	0.648	1,535	717	0.047^*^	9.06	3.79	0.086	0.63	0.688	0.014^*^
		4th	2065	970		1,355	490		7.77	1.72		0.216	0.479	
		5th	2,243	1,175		1,188	331		7.78	1.84		0.355	0.486	

Anova test (^*^p < 0.05).

In Table 4, regarding the LF words, we noticed that there was a difference between the groups for the variable Latency for the words of C1 (“bolha/bubble”; “tipo/type”); C2 (“flora/flora”; “ruga/wrinkle”; “saga/saga”; “flanco/flank”); and for C3 (“xadrez/chess”). There was also a difference for the movement duration variable for C1 words (“moto/motorcycle”; “tipo/type”); for all words in C2 and C3. For the variable movement fluency, there was no difference for the words of C1; there was a difference for most of the words in C2 (except “saga/saga “); and for all C3 words (except “xadrez/chess” and “chifre/horn”). For the gaze variable, there was no difference for words C1 and C2 words; and the words of C3 (“classe/class”; “concha/shell”; “chifre/horn “). To better verify such differences, a comparison was made between the *p* values, in order to verify which groups presented the comparisons, based on Tukey’s Multiple Comparison (*post hoc*; Table 5).

**Table 5:** Comparison of *p* values for variables between groups for LF words.

	Word	Group	Latency		Movement duration		Movement fluency		Gaze	
			3rd	4th	3rd	4th	3rd	4th	3rd	4th
C1	Bolha/Bubble	4th	0.001^*^		0.869		0.736		0.333	
		5th	<0.001^*^	0.684	0.468	0.734	0.287	0.663	0.216	0.937
	Tipo/type	4th	<0.001^*^		0.807		0.323		1	
		5th	<0.001^*^	1	0.177	0.404	0.234	0.96	0.204	0.157
C2	Flora/flora	4th	<0.001^*^		0.744		0.89		0.979	
		5th	0.002^*^	0.945	0.066	0.005^*^	0.043^*^	0.007^*^	0.208	0.106
	Regra/rule	4th	0.445		1		0.979		0.845	
		5th	0.215	0.843	0.01^*^	0.005^*^	0.029^*^	0.028^*^	0.48	0.166
	Ruga/wrinkle	4th	0.028^*^		0.892		0.812		0.504	
		5th	<0.001^*^	0.263	0.045^*^	0.086	0.024^*^	0.07	0.638	0.087
	Saga/saga	4th	0.035^*^		0.656		0.84		0.96	
		5th	0.032^*^	0.989	0.118	0.008^*^	0.268	0.065	0.598	0.725
	Flanco/flank	4th	0.181		0.997		0.901		0.921	
		5th	0.025^*^	0.57	0.048^*^	0.036^*^	0.05	0.091	0.921	1
C3	Chance/chance	4th	0.422		0.28		0.153		0.042^*^	
		5th	0.202	0.844	0.004^*^	0.118	0.003^*^	0.208	0.42	0.462
	Classe/class	4th	0.118		0.098		0.064		0.029^*^	
		5th	0.421	0.756	0.002^*^	0.259	0.001^*^	0.261	0.004^*^	0.659
	Concha/shell	4th	0.741		0.111		0.377		0.01^*^	
		5th	0.628	0.187	0.009^*^	0.476	0.035^*^	0.377	0.002^*^	0.801
	Xadrez/chess	4th	0.135		0.241		0.8		0.227	
		5th	0.041^*^	0.792	0.009^*^	0.263	0.068	0.184	0.047^*^	0.654
	Chifre/horn	4th	0.639		0.369		0.112		0.01^*^	
		5th	0.949	0.817	0.036^*^	0.393	0.137	1	0.143	0.555

Anova test, Tukey’s multiple comparison (*post hoc*; ^*^p < 0.05).

Table 5 indicated that, for the latency variable, there was a difference between 3rd and 4th, and between 3rd and 5th grade for words from C1 and C2, suggesting improvement in the phoneme-grapheme conversion mechanism for words that are independent of the context – that is, regardless of the spelling context, there is only one phoneme-grapheme relationship (rule C1) and those dependent of context (rule C2). However, for words from C3, only the word “xadrez/chess” showed a difference between 3rd and 5th grade, while the other words do not present differences regarding latency time. These findings suggest that for C3, the opacity of words implies a failure in access to orthographic lexicon and, consequently, delay to start the motor act of handwriting. Regarding the movement duration and Movement fluency variables, for the words from C2, it was noted that there was a difference between the 3rd and 5th and between 4th and 5th grade, suggesting a decrease in writing production time and disfluency, according to the advance in the school grade levels. These findings indicated that for C2, reading and writing practices may have influenced the development of the orthographic lexicon. For the words of C3, there was a difference for the Movement Duration and Movement fluency variables in the comparison between 3rd and 5th grade. There was also a difference for gaze variable. These findings suggest that there was a difficulty in formation of the orthographic lexicon for C3 words, considering that the student performed pauses to seek visual information of the word through gaze.

A comparison was made between the HF and LF words considering each writing coding rule. Although, word activation can experiment with different motor programs, it was possible to observe that there is a difference between the values, indicating that the students had greater difficulties in the words of LF (Table 6).

**Table 6:** Comparison of variables between HF and LF words.

				3rd grade			4th grade			5th grade		
				Mean	SD	Value of *p*	Mean	SD	Value of *p*	Mean	SD	Value of *p*
C1	Movement duration	Olho/eye	HF	1,360	884	0.012^*^	1,216	390	0.000^*^	931	324	0.001^*^
		Dama/lady	LF	869	437		879	367		702	173	
	Movement fluency	Olho/eye	HF	9	6	0.006^*^	8	2	0.000^*^	5	2	0.156
		Dama/lady	LF	5	2		5	2		5	1	
	Movement duration	Nome/Name	HF	1,160	593	0.045^*^	1,018	451	0.168	749	208	0.725
		Mapa/map	LF	883	376		889	338		728	264	
	Movement duration	Velha/old	HF	1,336	502	0.027^*^	1,262	414	0.042^*^	990	254	0.000^*^
		Moto/motorcycle	LF	1,016	538		1,015	593		738	184	
	Movement duration	Vida/life	HF	1,405	814	0.024^*^	1,149	482	0.044^*^	946	278	0.375
		Tipo/type	LF	1,011	347		971	215		892	188	
	Movement disfluency	Vida/life	HF	7	4	0.000^*^	6	3	0.000^*^	5	1	0.000^*^
		Tipo/type	LF	2	2		2	1		2	1	
C2	Movement duration	Lobo/Wolf	HF	9,695	4,843	0.000^*^	8,977	2,960	0.000^*^	8,451	3,597	0.000^*^
		Flora/flora	LF	1,398	558		1,389	492		1,098	360	
	Movement disfluency	Lobo/Wolf	HF	9.36	1.44	0.000^*^	2.90	1.22	0.000^*^	2.37	1.62	0.000^*^
		Flora/flora	LF	8	3		8	2		7	1	
	Gaze	Lobo/Wolf	HF	0.04	0.192	0.000^*^	0.08	0.277	0.003^*^	0.00	0.000	0.000^*^
		Flora/flora	LF	0.56	0.698		0.49	0.768		0.48	0.626	
	Latency	Gato/cat	HF	2099	762	0.004^*^	1806	1,001	0.455	1715	624	0.161
		Regra/rule	LF	3,465	2,251		1961	768		2077	1,279	
	Movement duration	Gato/cat	HF	1,417	591	0.031^*^	1,105	402	0.483	844	195	0.674
		Regra/rule	LF	1,104	437		1,178	493		865	193	
	Movement disfluency	Gato/cat	HF	9	3	0.047^*^	7	3	0.399	5	1	0.028
		Regra/rule	LF	7	2		7	2		6	1	
	Movement duration	Tempo/time	HF	1,313	618	0.133	1,161	406	0.380	927	225	0.008^*^
		Ruga/wrinkle	LF	1,080	494		1,080	381		792	161	
	Movement disfluency	Mundo/world	HF	8	5	0.238	7	3	0.083	5	1	0.047^*^
		Saga/saga	LF	6	3		6	2		5	1	
C3	Movement duration	Casa/house	HF	1,067	567	0.053	1,054	382	0.087	770	246	0.001^*^
		Chance/chance	LF	1,429	761		1,228	471		976	227	
	Movement disfluency	Casa/house	HF	7	4	0.041^*^	7	2	0.050	5	1	0.000^*^
		Chance/chance	LF	9	5		8	2		7	1	
	Gaze	Casa/house	HF	0.04	0.192	0.000^*^	0.03	0.164	0.001^*^	0.00	0.000	0.000^*^
		Chance/chance	LF	0.67	0.620		0.32	0.475		0.48	0.570	
	Gaze	Coisa/thing	HF	0.19	0.396	0.001^*^	0.11	0.393	0.036	0.00	0.000	0.004^*^
		Classe/class	LF	0.63	0.492		0.32	0.475		0.23	0.425	
	Movement duration	Bruxa/witch	HF	1,263	602	0.567	1,118	388	0.564	853	195	0.002^*^
		Xadrez/chess	LF	1,356	582		1,176	466		1,008	189	
	Movement disfluency	Bruxa/witch_V	HF	8	3	0.707	7	2	0.034^*^	6	1	0.000^*^
		Xadrez/chess	LF	8	4		8	3		7	1	
	Gaze	Bruxa/witch	HF	0.11	0.320	0.000^*^	0.05	0.229	0.000^*^	0.00	0.000	0.000^*^
		Xadrez/chess	LF	0.78	0.641		0.54	0.558		0.42	0.502	
	Movement duration	Bicho/animal	HF	1,369	747	0.409	1,222	545	0.274	948	221	0.001^*^
		Chifre/horn	LF	1,535	717		1,355	490		1,188	331	
	Movement disfluency	Bicho/animal	HF	8	5	0.436	7	3	0.489	6	1	0.000^*^
		Chifre/horn	LF	9	4		8	2		8	2	
	Gaze	Bicho/animal	HF	0.15	0.362	0.002^*^	0.03	0.164	0.026^*^	0.03	0.180	0.001^*^
		Chifre/horn	LF	0.63	0.688		0.22	0.479		0.35	0.486	

*T*-test, (^*^p < 0.05).

In Table 6, we note that for the C1 rule words, there was a significant difference between the HF and LF words for comparisons of duration and fluency, with a lower value being observed for the HF words. It was also observed that there was no difference between HF and LF for the gaze and latency variables, suggesting that regularity (rule C1) favored access to the lexicon for both HF and LF words.

As for the C2 rule words, there was a significant difference for the comparisons of duration between the HF-LF pairs (“lobo/wolf”-“flora/flora”; “gato/cat”-“regra/rule”; “tempo/time”-“ruga/wrinkle”) and for fluency between the HF-LF pairs (“lobo/wolf”-“flora/flora”; “gato/cat”; “mundo/world”-“saga/saga”), with a lower value being observed for the words of HF. It was also observed that there was a difference between HF and LF for gaze (“lobo/wolf”-“flora/flora”) and latency (“gato/cat”-“regra/rule”) variables. These findings suggest that both regularity and word frequency impacted movement. As for the C3 rule words, there was a significant difference for comparisons of duration between HF-LF pairs (“casa/house”- “chance/chance”; “bruxa/witch”-“xadrez/chess”) and for fluency between HF-LF pairs (“casa/house”-“chance/chance”; “bruxa/witch”-“xadrez/chess”; “bicho/animal”-“chifre/horn”). It was also observed that there was a difference between HF and LF for gaze variables (“casa/house”-“chance/chance”; “coisa/thing”-“classe/class”; “bruxa/witch” –“xadrez/chess”; “bicho/animal”- “chifre/horn”) and latency (“gato/cat”- “regra/rule”). There was no difference between latency values. These results suggest that increasing word complexity (C3 rules) impacted movement variables, but also increased the need to search for confirmation of how the word was spelled (gaze).

## 4. Discussion

This study presented an evaluation of the parameters of fluency and duration of movement, and latency time. There are no Brazilian studies with these measures, using technologies tool’s assessment. We chose to evaluate the variables separately considering the frequency of words, being for HF and LF words. Comparisons were performed within each coding rule of the Brazilian Portuguese writing system. Such procedures were taken in order to avoid different types of cascaded effects, as mentioned by Roux et al. (2013). The findings of this study indicated that there was a decrease in movement duration and movement fluency from 3rd to 5th grade for HF and LF words. Although these processes did not occur in the same way. Orthographic aspects had influenced the performance of kinematic variables. This can be noticed when we observe the difference performance for coding rules for HF and LF.

For HF words, there was a decrease in movement duration and movement fluency, for all coding rule (C1, C2 and C3), suggesting an improvement in the use of motor planes combined with the formation of orthographic lexicon. We can say that there was an influence of effect for HF words, which influenced performance, as they improved progressively from 3rd to 5th, since the increased exposure to words favored the establishment of the phoneme-grapheme relationship. International studies of writing motor development have indicated that movement duration and disfluency decrease between the ages of 8 and 9 years and become relatively stable by age 10 years (Meulenbroek and van Galen, 1990; Mojet, 1991; Zesiger et al., 1993). The decrease is mainly due to maturation and motor practice. In the same way, for Brazilian students, it was possible to notice that the decrease in the cognitive load of the writing movement favored the writing of HF words, indicating that writing practices favored the formation of long-term orthographic memory, suggesting an effect of lexicality for HF words. According with Shibata and Omura (2018), cognitive load is the amount of working memory in use to perform the task. As mentioned by Bonin et al. (2016), and Purcell et al. (2011), although there was a reduced spelling regularity effect. As mentioned by Kandel and Perret (2015a), when writing movements are fast and smooth, they require less sensory control and working memory. This results in a decrease in cognitive load. The consequence is that writing movements become automatized between ages 9 and 10.

Regarding LF words, the effect of orthographic regularity could be noticed. The students had improved progressively from 3rd to 5th grade fluency and duration of movement for words with coding rules of type C1 and C2. The same cannot be observed for C3. These findings suggest that the movements became automatic from the 3rd to the 5th grade, suggesting the impact of the effect of orthographic regularity and lexicality. This study revealed that lexical and sub-lexical activation affected motor production. For HF and LF words, lexical and sublexical process favored motor programming.

Still, the results of the comparison between HF and LF words indicated that the regularity of the words played an important role in lexical access, and the HF words could be accessed through the lexical route, especially for the C1 rule words, as highlighted by Caramazza (1988) and Afonso et al. (2018) lexical route gives access to the spelling of whole words from long-term memory so it would be used when spelling familiar words.

However, the results of the comparison between HF and LF words indicated that the regularity of the words played an important role in lexical access, and the HF words could be accessed through the lexical route, especially for the C1 rule words, which do not showed no difference between latency times and neither the need to use the gaze.

Still in the comparison between HF-LF words, in relation to the C2 rule words, we noticed that there is still a need to use lexical and sublexical routes, since for some words, it was necessary to use the eye to check the spelling of the word. This finding corroborates studies, which have already indicated that the lack of teaching based on the reflection of spelling rules, on the part of students, makes it difficult to appropriate spelling rules (Scliar-Cabral, 2003b; Germano and Capellini, 2011, 2019; Chiaramonte and Capellini, 2022).

Nonetheless, for the words of C3, the motor improvement did not prevent the cognitive overload resulting from the spelling conflict, related to central process. Collaborating with Olive and Kellogg (2002), unless automatic, the transcription processes can place so many demands on working memory that they interfere with other higher-order processes required for writing, such as planning and reviewing. That is, we noticed that there was a progression in the decrease in duration and disfluency for LF, however, such words require more systematic instruction from the school, that is, these effects were noticed due to the greater unpredictability of phoneme-grapheme correspondences, rule knowledge spelling and less opportunity to be exposed to these words impacted the students’ performance.

Döhla et al. (2018) also refers to the importance of working memory for the maintenance and manipulation of phonological information in order to access orthographic representations of writing, thus allowing the automation of handwriting, and the release of cognitive resources.

Combined with this, the students used the gaze as a support feature for checking the spelling of the word, that is, they became more dependent on visual clues from the word. These aspects suggest that there was an effect of orthographic regularity, and that the complexity and unpredictability of the C3 rule was not yet fully automatic, suggesting failure of long-term orthographic lexicon formation. When words are unfamiliar, such as those with LF of C3 rules, we can infer that students have not formed their orthographic representations (Perfetti et al., 1992; Share, 1999). Thus, to write this type of word, the student must memorize the spelling of the entire word and remember that there is a part of the word, such as the syllable that contains the spelling conflict, which will require more attention. (Kandel and Valdois, 2006). Therefore, the student must use strategies to be able to write the word without error, such as process letters separately, aiming to identify and locate letters that contain irregularities; or to write the word applying graphophonological conversion rules.

Nevertheless, irrespective the strategy used, students have made pauses while writing, verified in this study by the increased number of gaze, constituting additional cognitive loads that consume time and result in increased processing time. Kandel and Valdois (2006) have shown that children program their handwriting movements according to the syllabic structure of the word, as orthographic syllabification for irregular words is not so easy as regular words. This could be the next step for research for Brazilian Portuguese language.

As pointed out by Kandel and Perret (2015a,b), and in accordance with our results, orthographically irregular words (C2 and C3 coding rules) required more processing demands than regular words (C1 coding rule), suggesting that handwriting movements were affected by central processes. This “regularity effect” has been documented in previous research in other language (Delattre et al., 2006; Roux et al., 2013), but still has not been documented in Brazilian Portuguese language.

Regarding latency, which refers to lexical access, we noticed that for the HF words, there was a decrease in latency, according to the progression from the 3rd to the 5th year, for the three coding rules. This finding suggests that the HF of the word favored the recovery and access of the phoneme-grapheme conversion mechanism, indicating that there was a long-term memorization of words in the orthographic lexicon (Kandel and Valdois, 2006).

Conversely, for LF words, it was possible to notice that the lexical process and orthographic regularity influenced the students’ performance. For the latency of the words of C1 and C2, there was a decrease in the access time for students, with the progression from 3rd to 5th grade. However, for C3 words, most words did not differ in latency time. This finding suggests failure in the formation of the long-term orthographic lexicon, especially for words with LF and greater unpredictability (C3), and subsequent need of longer time to start the writing movement. In the case of words with C3 rules, in the comparison between pairs of HF-LF words, the students possibly had to access the word through the phonological route that is, looking for possible phoneme-grapheme relationships in Brazilian Portuguese, being verified by the difference between the kinematic variables. Thus, due to the use of the sublexical route, and the spelling uncertainty (Central Processes), the students relied even more on visual feedback, increasing the need to look for spelling information on the screen (greater number of gaze in the comparison between the words of LF and HF). As mentioned by Caramazza (1988), the sublexical route or assembled route makes use of knowledge about the links between phonology and orthography and provides a phonologically plausible spelling for non-words or low-frequency words. Moreover, in accordance with Afonso et al. (2015), our findings had showed that phonology-to-orthography influenced word spelling.

Going further, we can infer that there was a regularity effect, regarding the importance of phoneme-grapheme mappings, as manifested by shorter latencies and writing durations for HF words. It is emphasized that, in situations where there is competition for the same phonetic context (rule C3), it is necessary to have metalinguistic knowledge, especially semantic and morphological knowledge, which can help in choosing the letter or grapheme that will represent it. Nonetheless, these rules are dependent on spelling lexical memory (Scliar-Cabral, 2003a). These findings indicate that the students had difficulties in the formation of the orthographic mental lexicon, which were aggravated by the lack of systematic teaching of conversion and by the lack of strategies aimed at the visual memorization of these words. (Germano and Capellini, 2019; Chiaramonte and Capellini, 2022).

We can also infer that the students maintained activated the central and peripheral process for these words, because they needed more time to access orthographic information and to program motor planes for handwriting. We also noticed a greater need to search for the word on the notebook screen, in order to confirm spellings aspects of the word. As mentioned in a study, typically developing children also showed that writing, pausing, and spelling are closely linked and that word writing can be influenced by word-level pause effects related to frequency and morphological complexity (Kandel et al., 2011).

We can also assume that the spelling of the words was processed before the beginning of the movement and during the production of the words, mainly for the words of LF and of greater irregularity (coding rule C3). It’s possible to assure this by observing the increase in latency and writing pauses (greater number of gaze). Unfortunately, as we do not perform letter-by-letter analysis – related with local aspects of movements – but of the entire word analysis, we can only infer that such motor programming of words may also have occurred in HF and regular words. In this way, this was a limitation of this study.

## 5. Conclusion

Despite being a pilot study, this one brought us many reflections and collaborations on the production of writing for Brazilian Portuguese. This study revealed that lexical and sub-lexical activation affected motor production. For HF words, we noticed that the lexical and sublexical process favored motor programming, that is, the central orthographic lexical memory cascaded the motor programs (peripheral processes), indicating an interaction between the central and peripheral processes, as in which maturation and school progression occurs. For LF words, we noticed that the lexical and sublexical process also impacted motor programming (peripheral processes). However, despite the maturation and school progression for the motor planes, there was an increase in latency time and in the need to search for word information, measured by the Gaze variable for words with greater irregularity (C3). Hence, LF and less predictable words demanded greater cognitive overload and, thus, a greater need for interaction between central and peripheral processes. Finally, this study provides further evidence that linguistic variables such as orthographic regularity and word familiarity affect in Brazilian Portuguese written language for handwriting performance.

## Data availability statement

The raw data supporting the conclusions of this article will be made available by the authors, without undue reservation.

## Ethics statement

The studies involving human participants were reviewed and approved by Research Ethics Committee of the Faculty of Philosophy and Sciences of São Paulo State University “Júlio de Mesquita Filho” (UNESP), Marilia, São Paulo, Brazil, under number CAAE: 87368618.4.0000.5406. Written informed consent to participate in this study was provided by the participants’ legal guardian/next of kin.

## Author contributions

GG designed the experiment and project, collected, and interpreted data. SC provided technical support and conceptual advice. All authors discussed the results and implications, contributed to writing the main manuscript, and approved the submitted version.

## Funding

We are grateful for the financing provided by The National Council for Scientific and Technological Development (CNPq) – Universal Process number 455208/2014-0, 2018.

## Conflict of interest

The authors declare that the present research was conducted in the absence of any commercial or financial relationships that could be construed as a potential conflict of interest.

## Publisher’s note

All claims expressed in this article are solely those of the authors and do not necessarily represent those of their affiliated organizations, or those of the publisher, the editors and the reviewers. Any product that may be evaluated in this article, or claim that may be made by its manufacturer, is not guaranteed or endorsed by the publisher.
